# Comparing the Invasibility of Experimental “Reefs” with Field Observations of Natural Reefs and Artificial Structures

**DOI:** 10.1371/journal.pone.0038124

**Published:** 2012-05-30

**Authors:** Katherine A. Dafforn, Tim M. Glasby, Emma L. Johnston

**Affiliations:** 1 Evolution and Ecology Research Centre, School of Biological, Earth and Environmental Sciences, University of New South Wales, Sydney, New South Wales, Australia; 2 New South Wales Department of Primary Industries, Port Stephens Fisheries Institute, Nelson Bay, New South Wales, Australia; Leibniz Center for Tropical Marine Ecology, Germany

## Abstract

Natural systems are increasingly being modified by the addition of artificial habitats which may facilitate invasion. Where invaders are able to disperse from artificial habitats, their impact may spread to surrounding natural communities and therefore it is important to investigate potential factors that reduce or enhance invasibility. We surveyed the distribution of non-indigenous and native invertebrates and algae between artificial habitats and natural reefs in a marine subtidal system. We also deployed sandstone plates as experimental ‘reefs’ and manipulated the orientation, starting assemblage and degree of shading. Invertebrates (non-indigenous and native) appeared to be responding to similar environmental factors (e.g. orientation) and occupied most space on artificial structures and to a lesser extent reef walls. Non-indigenous invertebrates are less successful than native invertebrates on horizontal reefs despite functional similarities. Manipulative experiments revealed that even when non-indigenous invertebrates invade vertical “reefs”, they are unlikely to gain a foothold and never exceed covers of native invertebrates (regardless of space availability). Community ecology suggests that invertebrates will dominate reef walls and algae horizontal reefs due to functional differences, however our surveys revealed that native algae dominate both vertical and horizontal reefs in shallow estuarine systems. Few non-indigenous algae were sampled in the study, however where invasive algal species are present in a system, they may present a threat to reef communities. Our findings suggest that non-indigenous species are less successful at occupying space on reef compared to artificial structures, and manipulations of biotic and abiotic conditions (primarily orientation and to a lesser extent biotic resistance) on experimental “reefs” explained a large portion of this variation, however they could not fully explain the magnitude of differences.

## Introduction

Habitat modification and invasive species are widely acknowledged to have detrimental impacts on native communities [1] and the two disturbances are likely to interact. The loss or modification of habitat can create windows of opportunity for the introduction of non-indigenous species (NIS) [2]. Their subsequent establishment and persistence will depend on properties of both the invading species and the ability of the recipient community to resist invasion [3], [4]. Biotic resistance of the resident species to arriving invaders can arise through competition, predation, herbivory and disease, and is mediated by local abiotic conditions that can act as stressors on either the resident species or the invader [5]. Field studies investigating biotic resistance have tended to focus on a single process, generally competition (up to 70% of field studies on plant invasion [5]), while few have investigated multiple processes or examined interactive effects of abiotic conditions.

Biotic control of invasibility may occur in communities where native herbivores graze the invading species [6] or where densities or abundances of invaders are reduced by native predators [7]. Biotic interactions will often be species-specific, but in some cases more diverse native communities can enhance invasion resistance because competition for resources in the community is greater [3], [8]–[10]. Diversity-invasibility research has increasingly sought to understand the role of functional diversity and identity in invasibility [4]. Functional groups of species perform similar ecological functions irrespective of their taxonomic relatedness [11]. Disturbances can release resources and shift competitive interactions in favour of exotic species [12], but can also negatively impact both native and non-indigenous species [13]. Invasion success will also be related to the functional components of the receiving community [4].

Anthropogenic disturbances play important roles in releasing resources for invading species by physical removal or modification of the resident assemblage (e.g. logging forests, plowing grasslands and trawling seabeds) [14]–[16]. Exotic plant infestations have been strongly associated with the creation of new habitat along vehicle tracks [17]. Similarly, gaps in seagrass beds caused by dredging or anchoring in the Mediterranean have been colonised by the invasive alga *Caulerpa taxifolia* [18]. Where gaps are not maintained they are likely to be quickly re-colonised by vegetative growth or recruitment of the surrounding organisms, often resulting in invaders being outcompeted [19], [20].

Anthropogenic activities not only influence the strength of biotic resistance to invasion, they often result in the creation of habitat with novel abiotic conditions that are exploited by invaders. For example, the White-throated Swift relies on cliffs as nesting sites in its native range, but has been able to utilize high-rise buildings to increase its urban invasive range [21] and there are other examples in the literature of NIS exploiting urban structures such as bridges and lamp posts as habitat [22]. Similarly, the invasive alga *Codium fragile* ssp. *fragile* (Suringar) Hariot utilizes rocky reef in its native range, but has been able to invade and disperse using human-constructed breakwalls [23], [24]. The construction of coastal defence and port infrastructure (e.g. pilings, pontoons and seawalls) has introduced novel habitats into the marine environment, which support distinct assemblages [25]–[27], and have an elevated abundance of exotic species [24], [28], [29]. Artificial structures may in fact act as ‘stepping stones’ [30] or ‘corridors’ [24] for the spread of NIS into natural communities, and association with artificial structures has been considered an identifying characteristic of marine NIS [31]. Wharves and marinas are becoming increasingly necessary to support marine infrastructure and therefore it is important to understand the ecological implications of these anthropogenic modifications.

**Figure 1:**
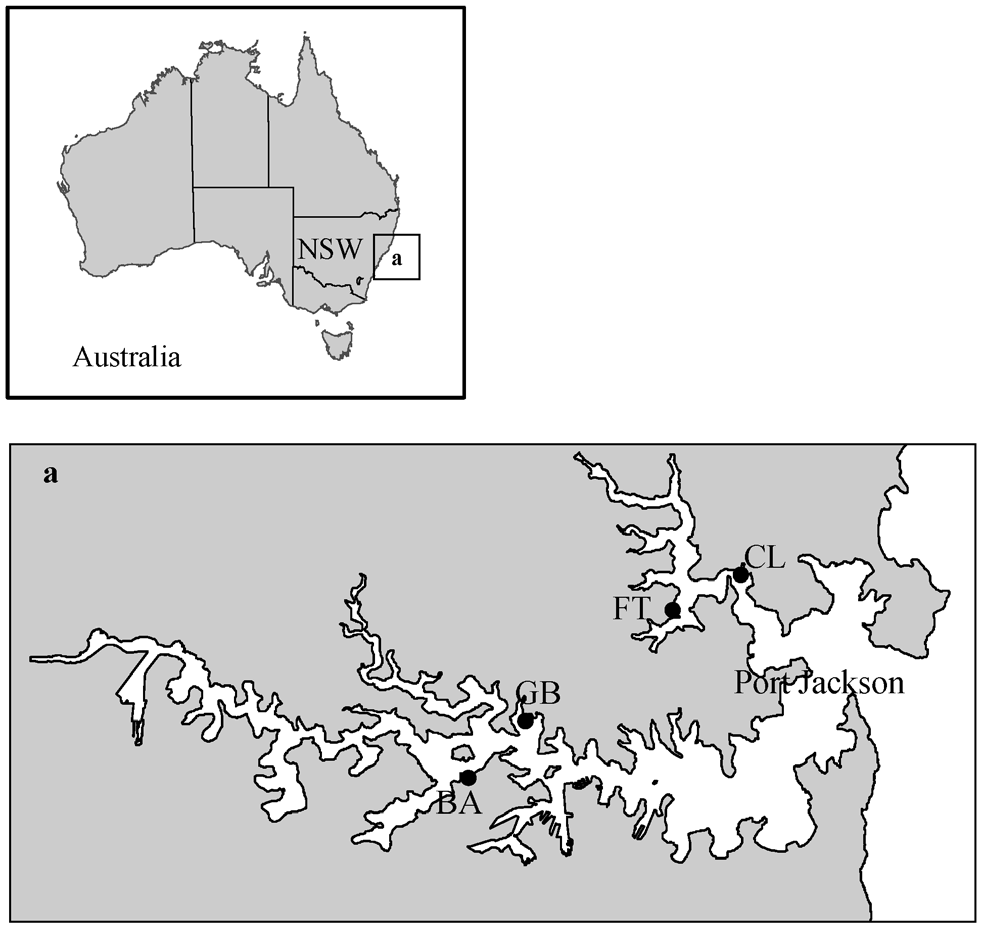
Study sites in Port Jackson, Australia. Sites were sampled in an *in-situ* underwater survey of vertical surfaces of artificial and natural habitats (BA, FT, GB and CL), photoquadrats of horizontal reef (BA, FT, GB) and sandstone plate deployment (FT). BA  =  Balmain, FT  =  Fig Tree, GB  =  Gore Bay and CL  =  Clontarf.

**Figure 2:**
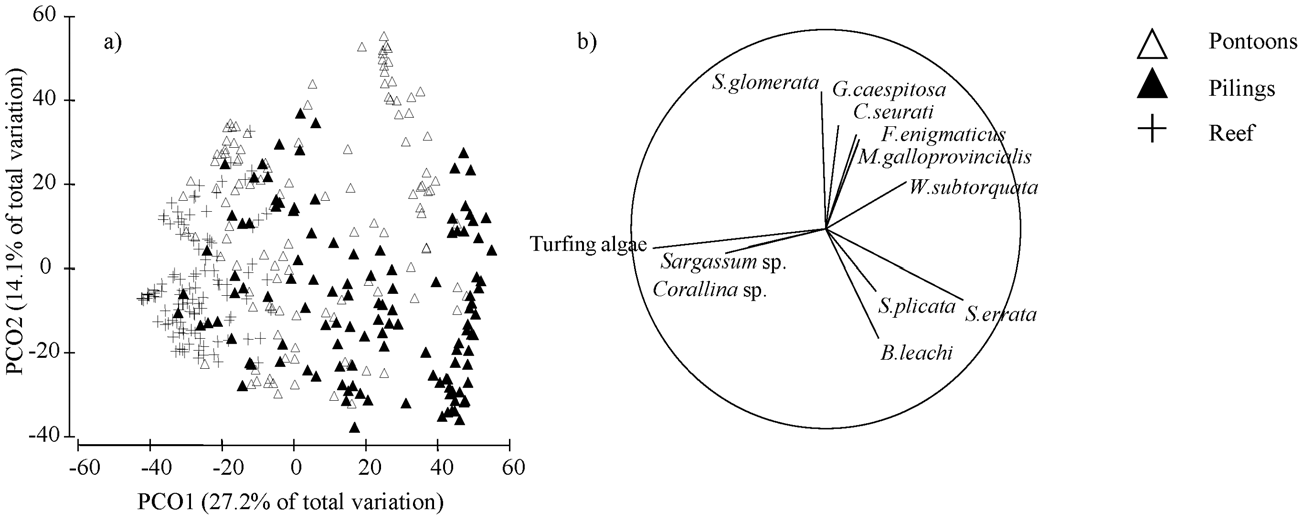
Principal co-ordinates analysis (PCO) comparing the hard-substrate community composition between artificial and natural habitats. Sampling was done on vertical surfaces of pilings, pontoons and rocky reefs at four sites in 2006 and 2007. The PCO plot (a) is coded by habitat and the vector overlay (b) indicates which taxa were positively correlated (>0.4) with the axes. The length of the vector indicates the strength of the relationship.

Despite the profusion of artificial structures in coastal areas, estuarine rocky reefs appear largely uninvaded. Environmental conditions (including increased light and sediment load) on horizontal rocky reefs are likely to contribute to sustained macroalgal dominance [32], [33]. Diverse invertebrate assemblages can also be present on vertical surfaces of rocky reefs [34], [35], however they tend to be more abundant on deeper, outer estuarine or coastal reef walls while studies of shallow estuarine rocky reefs have found them to be dominated by coralline and filamentous algae on both horizontal and vertical surfaces [30]. Experimental shading of rocky reefs can shift this competitive interaction and result in the development of invertebrate assemblages [36]. Because many estuarine invaders are sessile invertebrates [37], shading has the potential to facilitate invasion, by reducing light and siltation rates.

Here we compare the distribution of marine NIS between artificial structures (specifically pilings and pontoons) and natural rocky reefs using underwater surveys. We hypothesised that NIS would be more abundant on artificial structures, particularly pontoons [28] than on natural reefs and more abundant on vertical than horizontal reefs [29]. We proposed that these differences were a consequence of biotic resistance (of established native assemblages), and that when first deployed in the water, artificial structures are bare surfaces that are rapidly colonised by NIS. Thus it was predicted that if space were available (with experimentally deployed blank sandstone plates), NIS would colonise more so than natives. We also tested the hypothesis that differing abiotic conditions between artificial structures and natural reefs might account for the increased invasibility of artificial structures by non-indigenous fouling species. We deployed sandstone plates on rocky reef to experimentally test factors including light and sedimentation (through experimental shading and orientation) that might explain the differential abundances of NIS between artificial structures and reefs. We hypothesised NIS would occupy more space on plates that were shaded or vertically oriented (providing similar settling conditions to the vertical sides of pilings and pontoons). We expected horizontal unshaded plates to have the least NIS because of increased sedimentation and light, resulting in conditions better suited to native algal assemblages.

## Methods

The study was conducted at four sites in Port Jackson, Australia (33°50′ S 151°22′ E; Fig. 1) between Apr 2006 – Dec 2008. Port Jackson is a highly urbanised estuary and its shores are lined with artificial structures interspersed with sandstone rocky reef outcrops. At the four study sites, subtidal rocky reef (∼0–5 m) is dominated by turfing algae (primarily *Corallina officinalis*, but some *Amphiroa* sp., *Champia* sp. and *Laurencia* sp.) and canopy-forming algae (*Ecklonia radiata* and *Sargassum vestitum*) on both horizontal and vertical surfaces (with non-vegetated soft sediments deeper than 5 m).

**Figure 3:**
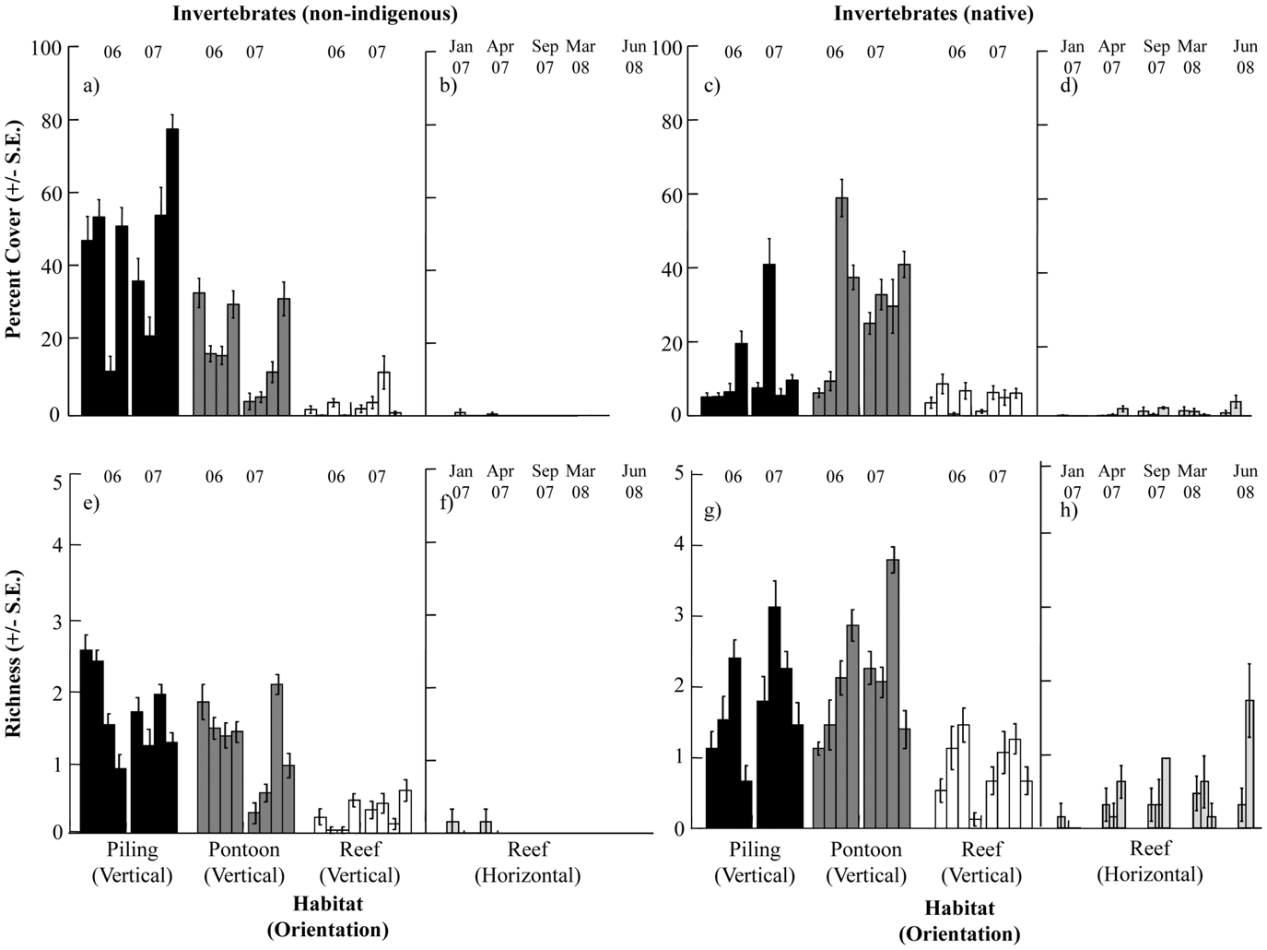
Percent cover and richness of invertebrates sampled on vertical surfaces of artificial and natural habitats and horizontal reef. Non-indigenous invertebrates were sampled from (a, e) vertical surfaces of habitats and (b, f) horizontal reef, native invertebrates were sampled from (c, g) vertical surfaces of habitats and (d, h) horizontal reef. In-situ sampling was conducted at four sites in 2006 and 2007. Individual bars represent sites ordered BA, FT, GB and CL from left to right. Sampling years (06/07) are indicated above the bars. Percent covers for horizontal reef were sampled in a separate survey at three sites at five sampling times (Jan 07, Apr 07, Sep 07, Mar 08, Jun 08). Individual bars represent sites ordered BA, FT, GB from left to right for each sampling time.

### Comparing Assemblages on Artificial and Natural Structures

We compared hard-substrate communities on artificial structures (fixed pilings and floating pontoons) and natural rocky reefs at four sites (structures and reefs were separated by <50 m at each site; Fig. 1). Pilings and pontoons were located at marinas and were constructed of wood (all pilings), concrete (pontoon – Balmain), fibreglass (pontoon – Clontarf, Gore Bay) and plastic (pontoon - Fig Tree). Information regarding the age and time since last cleaning was not available. However there was a well established fouling assemblage on all surfaces and no cleaning took place during the course of this study. Sampling was conducted twice; during the austral winters of 2006 and 2007. At each site, fifteen 0.25 m^2^ quadrats were randomly sampled *in situ* on scuba ∼1 m below MLWS on subtidal vertical surfaces (for consistency between structures and reefs). On pilings and pontoons, this was ∼2 m from the benthos and on reefs this was ∼0.5 m from the benthos. Percent cover in each quadrat was estimated using a grid of 36 regularly spaced points. Organisms within the quadrat, but not under a point were given a nominal value of 0.5%. Organisms were identified down to the lowest taxonomic level in the field and samples collected to confirm identities.

**Table 1:** PERMANOVA results comparing non-indigenous and native invertebrate species percent cover and richness sampled from artificial and natural habitats.

Source	df	SS	MS	Pseudo-F	P(perm)	SS	MS	Pseudo-F	P(perm)
			Percent cover				Species richness		
		a) Invertebrate (non-indigenous)				b) Invertebrate (non-indigenous)			
Habitat	2	210.95	105.47	23.25	**0.002**	44.59	22.29	16.48	**0.006**
Time	1	1.47	1.47	0.31	0.589	0.59	0.59	0.69	0.484
Site	3	6.76	2.25	6.00	**0.001**	0.63	0.21	1.66	0.180
HaxTi	2	19.42	9.71	2.94	0.129	4.61	2.30	2.80	0.143
HaxSi	6	27.23	4.54	12.09	**0.000**	8.12	1.35	10.62	**0.000**
TixSi	3	14.16	4.72	12.57	**0.000**	2.57	0.86	6.72	**0.000**
HaxTixSi	6	19.84	3.31	8.81	**0.000**	4.93	0.82	6.46	**0.000**
Res	336	126.15	0.38			42.78	0.13		
		**c) Invertebrate (native)**				**d) Invertebrate (native)**			
Habitat	2	89.36	44.68	20.57	**0.003**	16.60	8.30	21.58	**0.004**
Time	1	4.40	4.40	1.76	0.288	1.56	1.56	14.38	**0.042**
Site	3	21.94	7.31	17.57	**0.000**	8.61	2.87	17.79	**0.000**
HaxTi	2	0.90	0.45	0.14	0.871	0.48	0.24	0.39	0.699
HaxSi	6	13.03	2.17	5.22	**0.000**	2.31	0.38	2.39	**0.027**
TixSi	3	7.49	2.50	6.00	**0.001**	0.33	0.11	0.67	0.563
HaxTixSi	6	19.50	3.25	7.81	**0.000**	3.76	0.63	3.88	**0.001**
Res	336	139.86	0.42			54.19	0.16		

Non-indigenous invertebrate a) percent cover and b) species richness, native invertebrate c) percent cover and d) species richness compared between vertical surfaces of artificial (pilings and pontoons) and natural (reef) habitats. Habitats were sampled at twice at 4 sites. Significant results (p<0.05) are indicated in bold.

**Figure 4:**
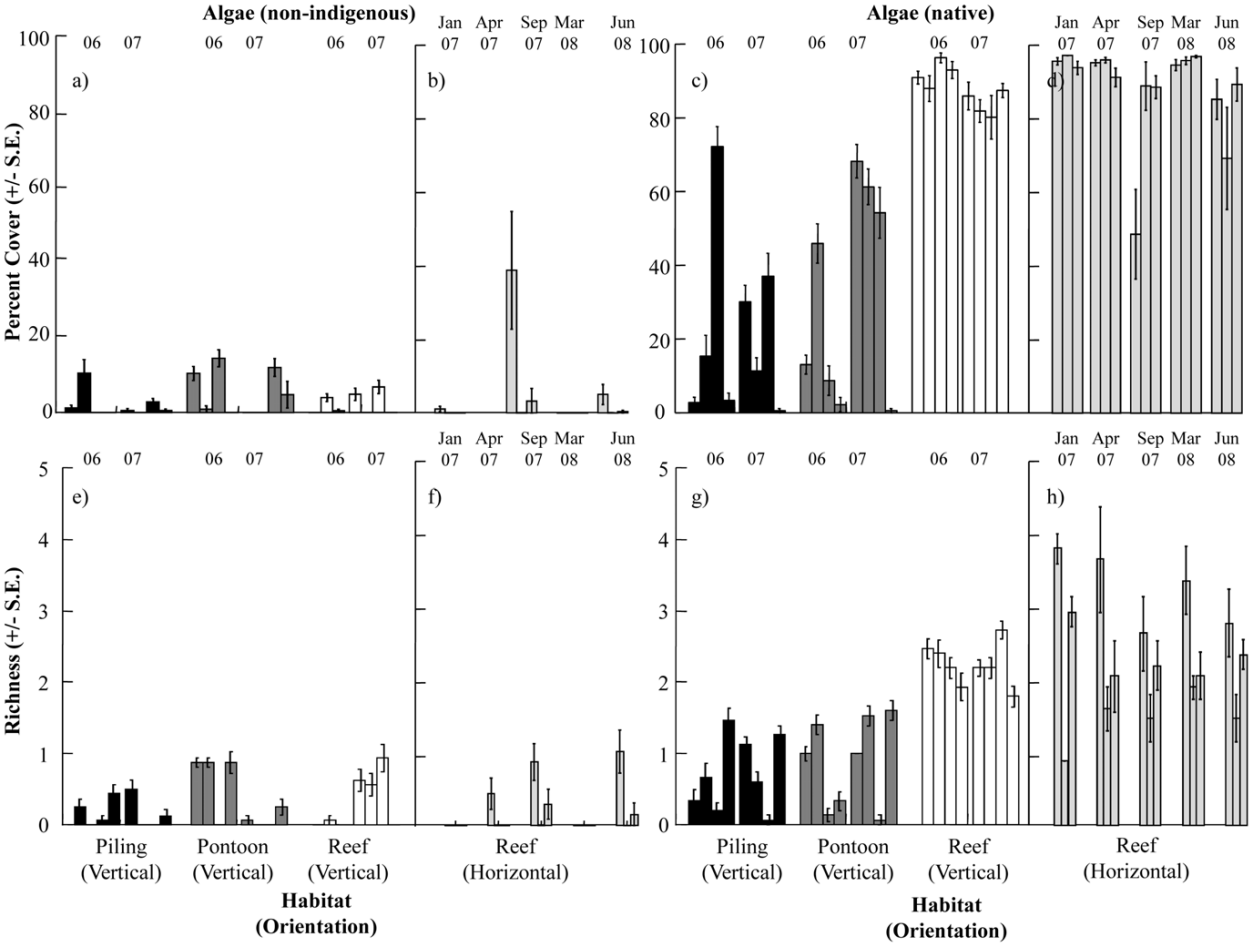
Percent cover and richness of algae sampled on vertical surfaces of artificial and natural habitats and horizontal reef. Non-indigenous algae sampled from (a, e) vertical surfaces of habitats and (b, f) horizontal reef and native algae sampled from (c, g) vertical surfaces of habitats and (d, h) horizontal reef. In-situ sampling was conducted at four sites in 2006 and 2007. Individual bars represent sites ordered BA, FT, GB and CL from left to right. Sampling years (06/07) are indicated above the bars. Percent covers for horizontal reef were sampled in a separate survey at three sites at five sampling times (Jan 07, Apr 07, Sep 07, Mar 08, Jun 08). Individual bars represent sites ordered BA, FT, GB from left to right for each sampling time.

**Table 2:** Pairwise comparisons of the non-indigenous and native invertebrate species percent cover and richness sampled from artificial and natural habitats.

Pairwise	Site (Year)	Pairwise	Site (Year)
Percent cover		Species richness	
a) Invertebrate (non-indigenous)		b) Invertebrate richness (non-indigenous)	
*Ha x Ti x Si*		*Ha x Ti x Si*	
Pi >Po > Re	FT (06/07), CL(06)	Pi >Po > Re	BA (06)
Pi = Po > Re	BA (06)	Pi = Po > Re	GB (06), FT (06/07), CL (07)
Pi >Po = Re	BA(07), CL(07), GB(07)	Pi >Po = Re	BA (07)
Po >Pi = Re	GB (06)	Po >Pi > Re	CL (06)
		Pi = Po, Po = Re, Pi > Re	GB (07)
Proportion where artificial > natural = **8/8**		Proportion where artificial > natural = **7/8**	
**c) Invertebrate (native)**		**d) Invertebrate richness (native)**	
*Ha x Ti x Si*		*Ha x Ti x Si*	
Po >Pi > Re	GB (06), FT (06/07), BA (07)	Po >Pi > Re	GB (06)
Po = Pi > Re	CL (07)	Po = Pi > Re	FT (06), BA (07)
Po = Pi, Pi = Re, Po > Re	BA (06), GB (06)	Po = Pi, Pi = Re, Po > Re	BA (06)
Pi = Po = Re	CL (06)	Pi = Po = Re	CL (06), GB (07)
		Pi >Po > Re	CL (07), FT (07)
Proportion where artificial > natural = **7/8**		Proportion where artificial > natural = **6/8**	

Non-indigenous invertebrate a) percent cover and b) species richness, native invertebrate c) percent cover and d) species richness compared between the vertical surfaces of artificial and natural structures. Habitat was the factor of interest in the comparisons and the direction of difference indicated in the left column. The sites and years at which these patterns occurred are indicated in the right column. Habitat  =  Pi (piling), Po (pontoon), Re (reef). Sites  =  BA (Balmain), CL (Clontarf), GB (Gore Bay) and FT (Fig Tree). Years  = 06 (2006) and 07 (2007).

We also surveyed the hard-substrate communities on horizontal reefs at three sites (BA, FT, GB; Fig. 1) with six 0.15 m^2^ photographic quadrats per site at five times (Jan 07, Apr 07, Sep 07, Mar 08, Jun 08). Quadrats were randomly positioned on the reefs 0.2–1 m below MLWS at each sampling time. Percent cover in each photoquadrat was estimated using a grid of 100 regularly spaced points superimposed over the photo. Organisms within the quadrat, but not under a point were given a nominal value of 0.5%. Sampling conducted on horizontal reef followed a different methodology to vertical reef because of the paucity of horizontal surfaces in our experimental area. Therefore instead of the extensive spatial replication used in the vertical sampling methodology, we increased the temporal sampling on the horizontal reefs. Surveys of horizontal reefs also form part of a larger study that examines the arrival and persistence of NIS on horizontal reefs (Dafforn et al., in prep).

**Table 3:** PERMANOVA results comparing non-indigenous and native algal species percent cover and richness sampled from artificial and natural habitats.

Source	df	SS	MS	Pseudo-F	P(perm)	SS	MS	Pseudo-F	P(perm)
			Percent cover				Species richness		
		a) Algae (non-indigenous)				b) Algae (non-indigenous)			
Habitat	2	10.90	5.45	1.22	0.348	2.35	1.17	0.79	0.488
Time	1	1.53	1.53	0.43	0.557	0.24	0.24	0.20	0.687
Site	3	10.36	3.45	11.55	**0.000**	3.08	1.03	10.28	**0.000**
HaxTi	2	50.72	25.36	9.87	**0.012**	16.82	8.41	11.37	**0.010**
HaxSi	6	26.87	4.48	14.97	**0.000**	8.91	1.48	14.88	**0.000**
TixSi	3	10.74	3.58	11.97	**0.000**	3.55	1.18	11.87	**0.000**
HaxTixSi	6	15.42	2.57	8.59	**0.000**	4.44	0.74	7.42	**0.000**
Res	336	100.49	0.29906			33.52	0.10		
		**c) Algae (native)**				**d) Algae (native)**			
Habitat	2	192.31	96.16	5.75	**0.045**	24.91	12.45	4.75	0.060
Time	1	9.04	9.04	1.71	0.295	1.24	1.24	2.33	0.248
Site	3	92.49	30.83	104.07	**0.000**	15.12	5.04	74.17	**0.000**
HaxTi	2	11.24	5.62	1.20	0.363	0.66	0.33	0.36	0.717
HaxSi	6	100.37	16.73	56.47	**0.000**	15.72	2.62	38.55	**0.000**
TixSi	3	15.84	5.28	17.82	**0.000**	1.60	0.53	7.85	**0.000**
HaxTixSi	6	28.09	4.68	15.81	**0.000**	5.51	0.92	13.51	**0.000**
Res	336	99.53	0.30			22.83	0.07		

Non-indigenous algal e) percent cover and f) species richness, native algal g) percent cover and h) species richness compared between vertical surfaces of artificial (pilings and pontoons) and natural (reef) habitats. Habitats were sampled at twice at 4 sites. Significant results (p<0.05) are indicated in bold.

**Table 4:** Pairwise comparisons of the non-indigenous and native algal species percent cover and richness sampled from artificial and natural habitats.

Pairwise	Site (Year)	Pairwise	Site (Year)
Percent cover		Species richness	
a) Algae (non-indigenous)		b) Algae (non-indigenous)	
*Ha x Ti x Si*		*Ha x Ti x Si*	
Po > Re >Pi	BA (06), GB (06)	Re >Pi = Po	CL (07), FT (07)
Pi >Po = Re	CL (06), BA (07)	Pi = Po > Re	GB (06)
Re >Pi = Po	CL (07)	Pi = Po = Re	BA (07), GB (07), FT (06)
Pi = Po > Re	FT (07)	Po >Pi = Re	BA (06)
Pi = Po = Re	FT (06), GB (07)	Pi = Re >Po	CL (06)
Proportion where artificial > natural = **5/8**		Proportion where artificial > natural = **2/8**	
**c) Algae (native)**		**d) Algae (native)**	
*Ha x Ti x Si*		*Ha x Ti x Si*	
Re >Po >Pi	BA(06/07), CL(06/07)	Re >Po >Pi	BA (06), CL (07)
Re >Pi >Po	GB (06)	Re >Pi >Po	CL (06)
Re >Po = Pi	FT(06/07), GB(06)	Re >Po = Pi	FT (06/07), BA (07)
		Re = Pi >Po	GB (06)
		Re = Po >Pi	GB (07)
Proportion where artificial > natural = **0/8**		Proportion where artificial > natural = **0/8**	

Non-indigenous algal e) percent cover and f) species richness, native algal g) percent cover and h) species richness compared between the vertical surfaces of artificial and natural structures. Habitat was the factor of interest in the comparisons and the direction of difference indicated in the left column. The sites and years at which these patterns occurred are indicated in the right column. Habitat  =  Pi (piling), Po (pontoon), Re (reef).Sites  =  BA (Balmain), CL (Clontarf), GB (Gore Bay) and FT (Fig Tree). Years  = 06 (2006) and 07 (2007).

### Investigating Effects of Orientation, Shading and Biotic Resistance on Invasibility Using Experimental “Reefs”

To investigate factors affecting the hard-substrate community on artificial and natural structures we conducted a manipulative experiment using Hawkesbury sandstone settlement plates (0.15 m^2^, 2 cm thick) at one site (FT; Fig. 1). Hawkesbury sandstone is the dominant natural hard substratum in the region. We established three fully factorial treatments that resulted in 12 treatment combinations each with five replicate sandstone settlement plates (total n = 60). Treatments were (1) resident assemblage (established and new), (2) shading (shaded, shade control and unshaded) and (3) orientation (horizontal and vertical). ‘Established’ assemblages had developed on settlement plates deployed horizontally (upward facing) for 12 mo to maximise algal colonisation. After collection, plates were kept in sea water and redeployed within 2 h. ‘Established’ assemblages were similar to nearby horizontal reef assemblages and typically comprised between 60–90% cover of the brown alga *Sargassum vestitum* (widespread throughout Port Jackson) with an understorey of filamentous algae (could not be surveyed before deployment due to the dense algal canopy). ‘New’ assemblages were represented by bare sandstone plates. In November 2007, established and bare plates were oriented horizontally or vertically and attached to six aluminium frames (200×10×10 cm) and randomly allocated to the following shading treatments (Supporting Information Figure S1). Black and transparent Perspex roofs (35×45 cm with a 3 cm folded edge) were attached 30 cm above shaded and shade control plates, respectively. Black roofs were used to reduce light reaching the plates below, but by their design also reduced siltation. Transparent roofs were therefore included to act as procedural controls for differences in flow and siltation. Unshaded plates represented unmanipulated controls for the experimental treatments. Roofs were cleaned weekly to remove fouling and sediment. Frames were deployed ∼1 m below MLWS adjacent to natural rocky reefs for 8 mo (Fig. 1). Frames were weighted to the benthos (soft sediment) with a besser brick at each end.

**Figure 5:**
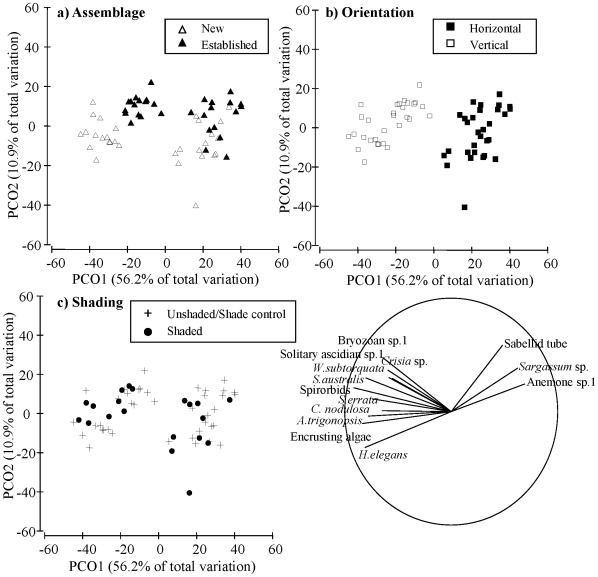
Principal co-ordinates analysis (PCO) comparing the hard-substrate community composition on experimental “reef” (sandstone plates). Points are are separated by a) resident assemblage (new or established), b) orientation (horizontal or vertical), and c) shading treatment (unshaded/shade control or shaded). The vector overlay indicates which taxa were positively correlated (>0.4) with the axes and the length of the vector indicates the strength of the relationship.

*Sargassum vestitum* formed a dense canopy over the established plate communities and was removed at the end of the experiment in order to census the understorey. The upward (horizontal) or outward (vertical) facing surfaces of plates were sampled live under a dissecting microscope by placing a grid of 100 points over the surface. Organisms on the plate, but not under a point were given a nominal value of 0.5%. To test the efficacy of the shading treatments, midday light was measured above two replicate plates of each shading treatment at each site for 28 d (Dec 2007) using a HOBO® data logger (Model UA-002-08; Onset Computer Corporation). Light meters were deployed above the plates and/or under shades (differences between orientations could not be compared). Sediment measurements were collected with 60 ml syringes from two replicate settlement plates in each treatment which allowed comparisons between resident assemblage, shading and orientation treatments. Sediment was ‘vacuumed’ *in-situ* from a randomly selected 1 cm^2^ area on the corner of each plate using the syringe. Each sample was filtered through a dried and pre-weighed 1 µm filter paper to obtain the fine fraction and then oven-dried (24 h at 70°C) and re-weighed.

Taxa identified during this study were classified as non-indigenous, native or cryptogenic according to the literature (Supporting Information Table S1). Turfing algae in this study included a complex dominated by *Corallina officinalis,* but with some *Champia viridis* and *Laurencia* sp. and encrusting algae were dominated by species of *Peyssonnelia.* Turfing and encrusting algae were grouped as native following classification in [29] which used the criteria from [31]. Exceptions to this were the brown algae *Colpomenia sinuosa* and *Dictyota dichotoma* which are non-indigenous [29].

**Table 5:** PERMANOVA results comparing the non-indigenous and native species percent cover and richness sampled from experimental “reef” (sandstone plates).

Source	df	SS	MS	Pseudo-F	P(perm)	SS	MS	Pseudo-F	P(perm)
			Percent cover				Species richness		
		a) Invertebrate (non-indigenous)				b) Invertebrate (non-indigenous)			
Assemblage	1	2.79	2.79	36.54	**0.000**	1.12	1.12	12.15	**0.001**
Orientation	1	18.32	18.32	239.69	**0.000**	8.76	8.76	94.64	**0.000**
Light	2	0.48	0.24	3.12	0.054	0.72	0.36	3.89	**0.027**
AsxOr	1	0.37	0.37	4.85	**0.036**	1.25	1.25	13.56	**0.001**
AsxLi	2	0.68	0.34	4.44	**0.016**	0.45	0.23	2.44	0.103
OrxLi	2	0.46	0.23	3.01	0.061	0.34	0.17	1.84	0.180
AsxOrxLi	2	0.37	0.19	2.43	0.100	0.47	0.24	2.56	0.090
Res	48	3.67	0.08			4.44	0.09		
		**c) Invertebrate (native)**				**d) Invertebrate (native)**			
Assemblage	1	49.15	49.15	6.37	**0.014**	0.10	0.10	0.59	0.443
Orientation	1	724.57	724.57	93.88	**0.000**	7.39	7.39	44.44	**0.000**
Light	2	96.21	48.11	6.23	**0.003**	0.22	0.11	0.67	0.519
AsxOr	1	45.60	45.60	5.91	**0.019**	0.01	0.01	0.07	0.796
AsxLi	2	9.30	4.65	0.60	0.557	0.05	0.02	0.14	0.876
OrxLi	2	75.20	37.60	4.87	**0.012**	0.17	0.09	0.52	0.595
AsxOrxLi	2	3.44	1.72	0.22	0.803	0.36	0.18	1.08	0.351
Res	48	370.47	7.72			7.98	0.17		
		**e) Algal (native)**				**f) Algal (native)**			
Assemblage	1	2.26	2.26	69.07	**0.000**	0.12	0.12	19.76	**0.000**
Orientation	1	0.04	0.04	1.28	0.260	0.01	0.01	2.15	0.147
Light	2	1.58	0.79	24.02	**0.000**	0.03	0.02	2.56	0.088
AsxOr	1	0.01	0.01	0.36	0.556	0.01	0.01	1.88	0.172
AsxLi	2	0.07	0.03	1.02	0.369	0.04	0.02	3.49	**0.035**
OrxLi	2	0.14	0.07	2.14	0.126	0.00	0.00	0.25	0.778
AsxOrxLi	2	0.07	0.03	1.01	0.378	0.01	0.00	0.62	0.553
Res	48	1.57	0.03			0.29	0.01		

Non-indigenous invertebrate a) percent cover and b) species richness, native invertebrate c) percent cover and d) species richness, and e) native algal percent cover and species richness. Analyses compared new and established assemblages on sandstone plates that were either oriented vertically or horizontally and subject to different shading treatments. Analyses were not conducted on non-indigenous algae because of low covers. Plates were collected and censused after 32 weeks. Significant results (p<0.05) are indicated in bold.

**Figure 6:**
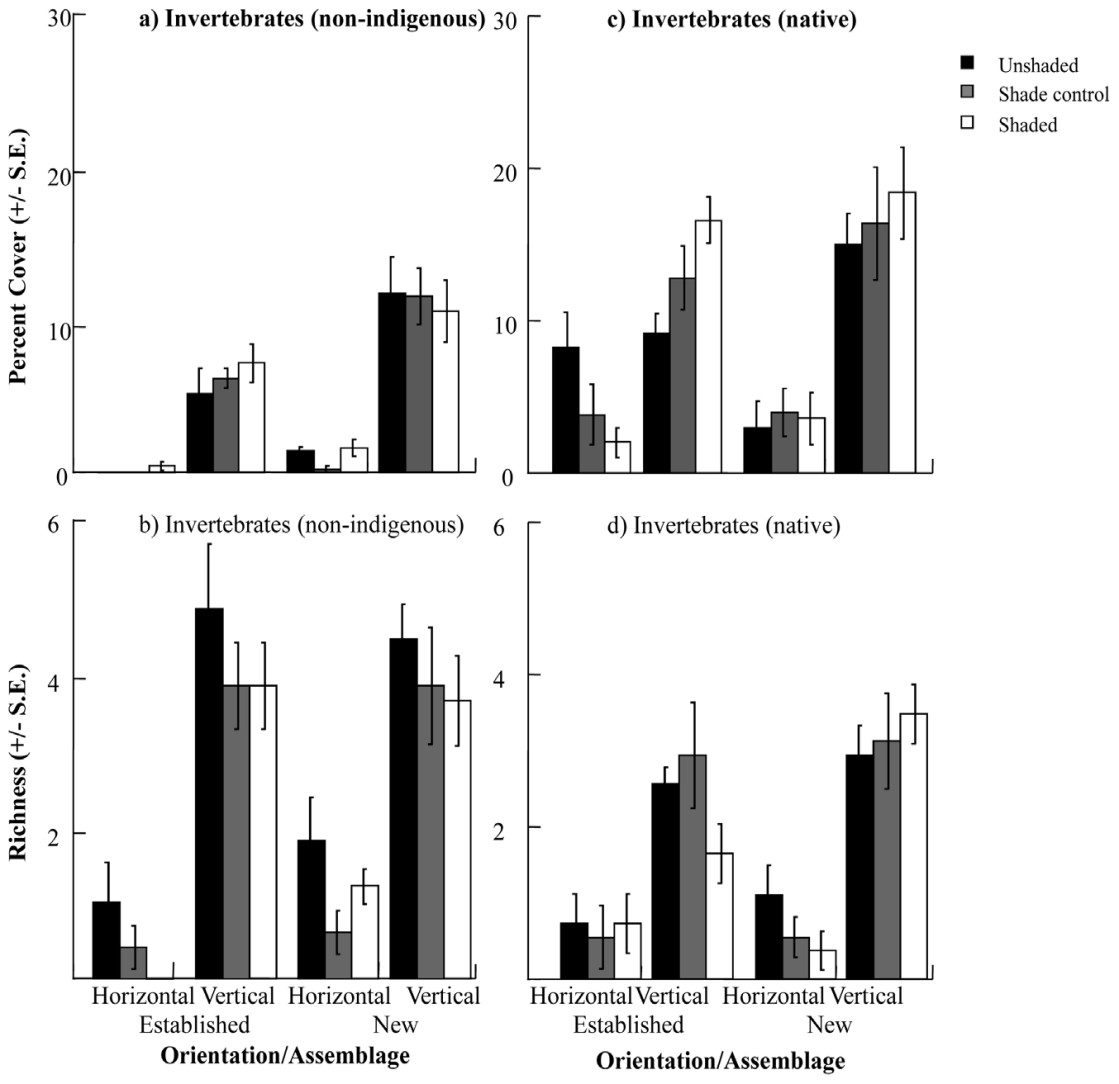
Percent covers and richness of invertebrates sampled on experimental “reef”. (a, b) non-indigenous invertebrates and (c, d ) native invertebrates were sampled on experimentally deployed sandstone plates. Bars are separated by orientation (horizontal or vertical) and assemblage (new or established) and coded for shading treatment (unshaded, shade control or shaded).

**Table 6:** Pairwise comparisons of the non-indigenous and native species percent cover and richness sampled from experimental “reef” (sandstone plates).

Pairwise Comparisons	Factor	Treatment	
**a) Non-indigenous invertebrate percent cover**			
*As x Or*	As	New	V > H
		Established	V > H
	Or	Vertical	N > E
		Horizontal	N > E
*As x Li*	As	New	US > SC
		Established	SH > US
	Li	Shaded	N > E
		Shade control	
		Unshaded	N > E
**b) Non-indigenous invertebrate richness**			
*AsxOr*	As	New	V > H
		Established	V > H
	Or	Vertical	
		Horizontal	N > E
**c) Native invertebrate percent cover**			
*As x Or*	As	New	V > H
		Established	V > H
	Or	Vertical	N > E
		Horizontal	
*Or x Li*	Or	Horizontal	
		Vertical	SH > SC
	Li	Shaded	V > H
		Shade control	V > H
		Unshaded	V > H
**d) Native invertebrate richness**			
*Or*			V > H
**e) Native algal percent cover**			
*As*			E > N
*Li*			US > SC > SH
**f) Native algal richness**			
*AsxLi*	As	New	SC = US > SH
		Established	
	Li	Shaded	E > N
		Shade control	E > N
		Unshaded	

Non-indigenous invertebrate a) percent cover and b) species richness, native invertebrate c) percent cover and d) species richness, and e) native algal percent cover and species richness. Analyses compared new and established assemblages on sandstone plates that were either oriented vertically or horizontally and subject to different shading treatments. Where an interaction term was significant, the assemblages, orientations or shading treatments for which these patterns were significant are indicated in the right column.

### Data Analyses

Hard-substrate community composition on the vertical surfaces of artificial structures and natural reefs were compared with a multivariate PERMANOVA and visualized with a Principal Components Ordination (PCO). For the in-situ survey, habitat (Ha) and time (Ti) were treated as fixed factors and site (Si) as a random factor. Non-indigenous and native invertebrate and algal percent covers were analysed separately with PERMANOVA. Data were 4^th^ root transformed and analyses conducted on a Bray-Curtis similarity matrix. Pairwise comparisons were conducted on significant results. Surveys of horizontal and vertical reefs were conducted at different times using different methodology. The use of photographic sampling of the fouling community may have underestimated local richness because of the two dimensional nature of a photograph. Hence, species richness data were not compared between the *in situ* quadrat sampling (vertical surfaces) and photographic sampling (horizontal surfaces) and we analysed frequencies to investigate differences in species distributions. Specifically, the frequency of occurrence of non-indigenous versus native invertebrates and algae was compared between our in-situ surveys (vertical reef, horizontal reef) using chi-squared tests of goodness of fit. This combined information on the number of species (NIS or native) and the number of replicates in which those species occurred and related this to what would be expected by chance given the available species pool.

**Figure 7:**
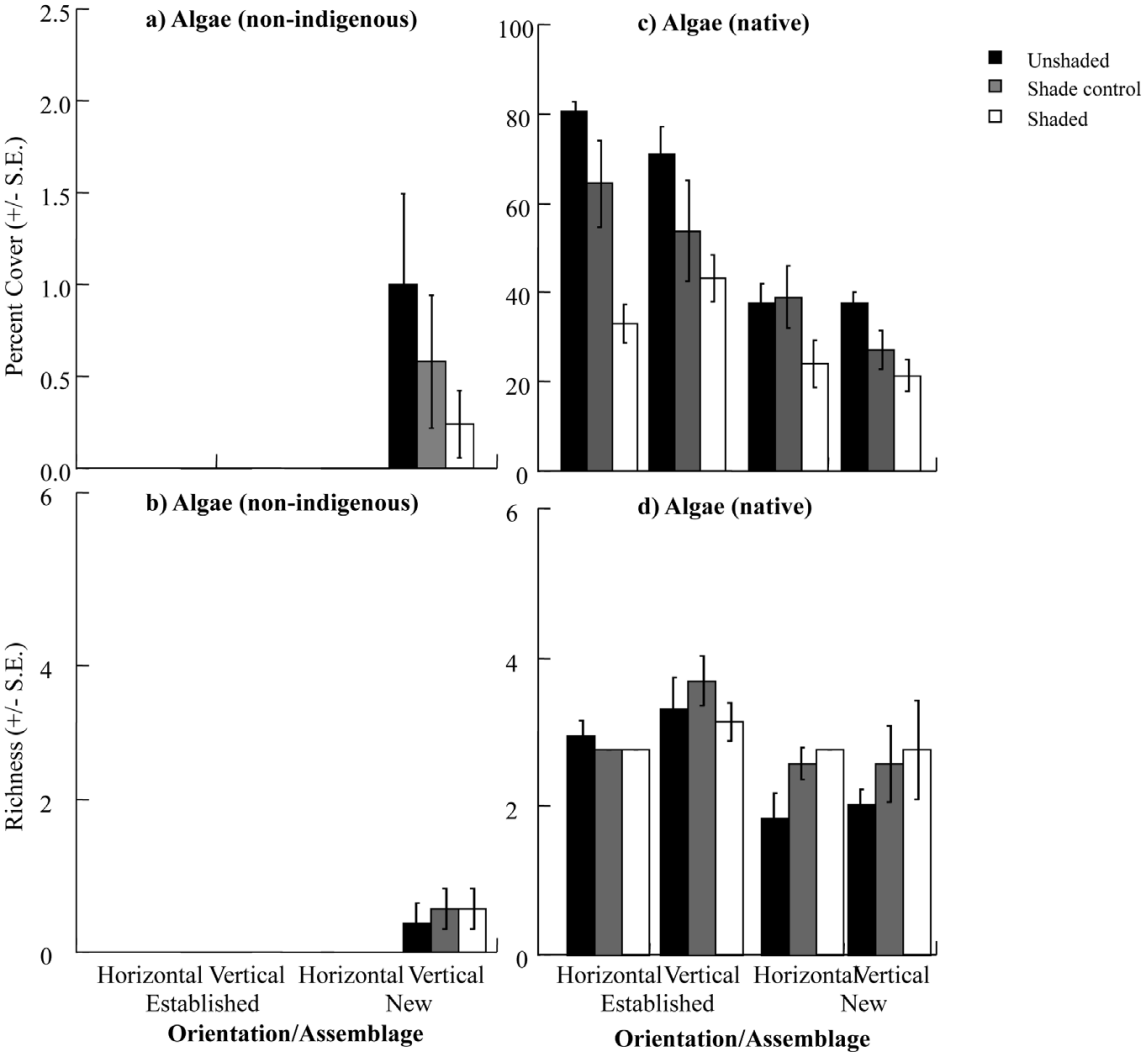
Percent covers and richness of algae sampled on experimental “reef”. (a, b) non-indigenous algae and (c, d) native algae were sampled on experimentally deployed sandstone plates. Bars are separated by orientation (horizontal or vertical) and assemblage (new or established) and coded for shading treatment (unshaded, shade control or shaded).

Hard-substrate community composition on the surfaces of sandstone plates were analysed with a multivariate PERMANOVA and visualized with a Principal Components Ordination (PCO). Data were 4^th^ root transformed and analyses conducted on a Bray-Curtis similarity matrix. Resident assemblage (As), shading (Sh) and substrate orientation (Or) were treated as fixed effects. Non-indigenous and native invertebrate and algal percent covers were analysed separately with PERMANOVA. Dry weight sediment data collected from the plates were analysed using a three-factor ANOVA (details as for sandstone plates). Light levels were also compared between shading treatments with a one-factor ANOVA. Pairwise comparisons were conducted on significant results. Correlations were used to compare native algal cover with covers of invertebrates (non-indigenous and native). Invertebrate (non-indigenous and native) and algal (native) covers were also compared to sediment loads and light levels. All analyses were performed using the PRIMER 6 statistical package with the PERMANOVA+ add-on (PRIMER-E, Plymouth Marine Laboratory, UK).

## Results

Sixty-five taxa were sampled during the entire study (most to genus or species). These included species of encrusting and arborescent bryozoans, solitary and colonial ascidians, and serpulid polychaetes. Twenty-two species were classified as non-indigenous, twenty-five as native and eighteen as cryptogenic or unidentified [38] (Supporting Information Table S1). Cryptogenic and unidentified species had very low average covers (0.01%–3.2%, Supporting Information Table S2) and were therefore included in the community composition analyses, but not analysed as a separate ‘cryptogenic’ group.

**Figure 8:**
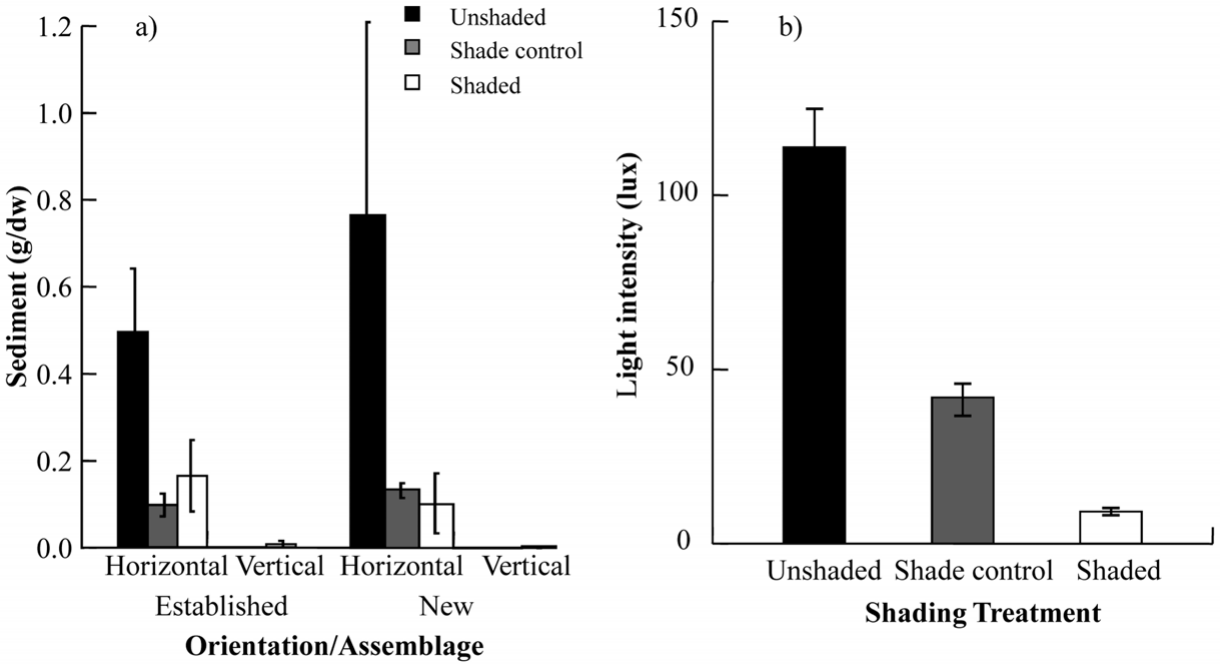
Sediment and light intensity collected during deployment of experimental “reef”. (a) Sediment (g/dry weight) was collected from individual plates prior to final collection of plates and (b) light (lux) was sampled above the plates with loggers for 28 days during the experiment. The averages are presented for each measure and bars are separated by (a) orientation (horizontal or vertical) and assemblage (new or established) and (a/b) coded for shading treatment (unshaded, shade control or shaded).

### Comparing Assemblages on Artificial and Natural Structures

Hard-substrate community composition differed significantly between artificial and natural structures at different sampling times and sites (Ha x Ti x Si: p<0.01, Fig. 2a). Although there was temporal (time) and spatial (sites) variation in the assemblages, community composition differed significantly among pilings, pontoons and reefs at all times and sites (pairwise comparisons, p<0.01). Estimates of components of variation revealed that habitat explained most of these differences (S = 741). Non-indigenous and native invertebrate percent covers and richness differed significantly between habitats (Fig. 3, Table 1). Both groups occupied less space (<10%) than native algae (∼80–100%) on vertical reefs (Fig. 3a,c & 4c). Non-indigenous invertebrate percent cover was 10–80% greater on pilings (*Styela plicata*, *Schizoporella errata* and *Botrylloides leachi*; Fig. 2b) and/or pontoons (*Ficopomatus enigmaticus*, *Watersipora subtorquata* and *Conopeum seurati*; Fig. 2b) than vertical reefs, but tended to be greatest on pilings (Fig. 3a, Table 2a). Non-indigenous invertebrate richness was also greater on artificial structures than reef at more than 80% of sites/times (Fig. 3e, Table 2b). Similarly, cover and richness of native invertebrates were generally greater on artificial structures (pilings or pontoons) than vertical reefs (Fig. 3c,g, Table 2c,d). Native invertebrates (*Saccostrea glomerata*, *Galeolaria caespitosa* and *Mytilus galloprovincialis planulatus*; Fig. 2b) tended to occupy the most space on pontoons, but this varied spatially and temporally with covers often similar between pontoons and pilings (Fig. 3c, Table 2c). Invertebrates (non-indigenous or native) occurred equally often on all vertically oriented habitats (pilings: χ^2^ = 0.45, p>0.05, pontoons: χ^2^ = 0.46, p>0.05, reefs (V): χ^2^ = 3.56, p>0.05). In contrast, horizontal reefs were largely uninvaded and native invertebrate species occurred around three times as often as non-indigenous invertebrates (χ^2^ = 12.74, p<0.001).

Non-indigenous and native algal percent covers and richness differed significantly between habitats (Fig. 4, Table 3). Non-indigenous algae (*Colpomenia sinuosa* and *Dictyota dichotoma*) occupied less space (<20%) on all structures than native algae and covers were typically greater on pilings or pontoons than on reefs (Fig. 4a,c, Table 4a). The greatest cover of non-indigenous algae occurred on horizontal reef at one time and one site (Fig. 4b) and this pattern was driven by *Colpomenia sinuosa*. Non-indigenous algal richness was low in all habitats (Fig. 4e,f) and did not show consistent patterns of difference between pilings, pontoon and reef (Table 4b). Native algae (turfing algae, *Sargassum vestitum* and *Corallina officinalis*) were relatively more abundant on vertical reefs than on pilings or pontoons (Fig. 2b). Native algal cover was more variable between sites and over time on artificial structures (pilings and pontoons) than on reefs and patterns for algae were similar between vertical and horizontal reefs (Fig. 4c,d, Table 4c). Patterns of native algal richness matched those of percent covers (Fig. 4, Table 4d). Native algae dominated space on horizontal reefs (Fig. 4d) while non-indigenous algal cover was much lower and variable (∼15% at a single site and almost absent from other sites) (Fig. 4b). Native algae occurred 2–7 times more often than non-indigenous algae in all habitats (pilings: χ^2^ = 12.52, p<0.001, pontoons: χ^2^ = 5.39, p<0.05, reefs (V): χ^2^ = 27.79, p<0.0001 and reefs (H): χ^2^ = 28.78, p<0.0001).

### Effects of Orientation, Shading and Resident Assemblage on Invasibility

Hard-substrate community composition on the sandstone plates differed significantly according to orientation, initial assemblage type and level of shading (Or x As x Li: p<0.01, Fig. 5). Estimates of components of variation revealed that orientation explained most of these differences (S = 1150), followed by the initial assemblage (S = 208). Invertebrates (native and non-indigenous) were most abundant on vertical plates regardless of the assemblage present (Fig. 5). The clearest differences related to initial assemblage were related to the relatively greater cover of native *Sargassum vestitum* on established horizontal plates (∼35%) compared to new horizontal plates (∼12%) (Fig. 5). Tubes of the polychaete worm *Chaetopterus* sp. and the anemone *Aiptasia* sp. also occupied more space in established horizontal assemblages than in other treatments, but this constituted only ∼4% and ∼2% cover respectively. *Schizoporella errata*, *Celleporaria nodulosa*, *Hydroides elegans* and encrusting algae occupied the most space in the new assemblages on the vertical plates, while *Watersipora subtorquata*, *Salmacina australis*, *Crisia* sp., *Scruparia* sp. and *Ascidiella aspersa* occupied relatively more space in the established assemblages on the vertical plates compared to other treatments (Fig. 5). Community composition also differed between unshaded/shade control and shaded plates (Fig. 5), however there were no clear patterns for species covers between treatments (pairwise comparisons, p>0.05).

Non-indigenous invertebrate cover differed with orientation, but also with resident assemblage and shading treatment (Table 5a). Similar to overall community composition, estimates of components of variation revealed that orientation explained most of these differences (S = 0.608), followed by the initial assemblage (S = 0.091). Vertical orientation increased the cover and richness of non-indigenous invertebrates (Fig. 6a,b, Table 6a,b) as did the absence of a biotic assemblage at the beginning of the study (percent cover only, Fig. 6a, Table 6a). Hence, the greatest richness and cover of invertebrate NIS occurred on vertical assemblages. Cover also varied with shading treatments in different assemblages, but the patterns were inconsistent between orientation (Table 6a), potentially related to small differences caused by shading treatments on new horizontal surfaces. Native invertebrate richness and percent covers differed significantly with assemblage, orientation and shading treatment (percent cover only, Table 5c,d). Covers and richness were always greater on vertical than horizontal plates regardless of assemblage or shading treatment (Fig. 6c,d, Table 6c,d). On horizontal plates, native invertebrate covers did not differ between new and established assemblages or between light treatments. In contrast, on vertical plates covers were greatest on established plates and those that were shaded (Fig. 6c, Table 6c). Invertebrates occupied less space in general than native algae (natives 10–20% and non-indigenous 5–10%) and invertebrate (non-indigenous and native) covers were negatively related to algal (native) covers (r = −0.341, p>0.05 and r = −0.126, p>0.05 respectively).

The non-indigenous alga *Colpomenia sinuosa* was found only on vertically oriented plates that had no resident assemblage at the beginning of the experiment (new; Fig. 7a). Cover and richness of native algae was (unsurprisingly) greater in established than new assemblages and greater on unshaded than shaded plates (Fig. 7c,d, Table 5e,f, 6e,f). Similar to surveys of horizontal and vertical reefs, native algal covers did not differ between horizontally and vertically oriented plates (Fig. 7c, Table 5e).

Sediment loads varied with orientation and with shading treatment (Fig. 8a, Or x Sh: F_2,12_ = 14.904, p<0.01). Sediment loads measured directly on plates were up to three times greater on horizontal than vertical plates and the presence of Perspex roofs on shaded and shade control treatments reduced sediment (∼60–75%) compared to unshaded treatments, but this was significant only on horizontal plates (pairwise test, p<0.05). Invertebrate (non-indigenous and native) covers were significantly negatively related to sediment loads on the plates. Non-indigenous invertebrates covers were reduced to <2% when just 0.1 g/dw sediment was present (r = −0.752, p<0.05). Native invertebrate covers appeared similarly impacted by sediment load, but covers were up to ∼8% when 0.6 g/dw sediment was present (r = −0.642, p<0.05). Native algal covers were weakly related to sediment loads, but there was a slight positive trend (r = 0.277, p>0.05).

Light levels also differed between shading treatments (Fig. 8b, Sh: F_2,165_ = 58.04, p<0.01). Logistical constraints meant that light differences between orientations could not be compared. Shaded plates received only 20% of the light received by unshaded plates (Fig. 6b). Light levels on the shade control plates were significantly lower than the unshaded plates, but still significantly more than on the shaded plates (pairwise test, p<0.01). There were slight trends for invertebrate (non-indigenous and native) covers to decrease with increasing light levels, while algae (native) increased, however these relationships were not significant (r = −0.189, p>0.05, r = −0.268, p>0.05 and r = 0.401, p>0.05 respectively).

## Discussion

Habitat modification is identified as a major threat to biodiversity and is a driver of invasion success in many systems [39]. The addition of artificial structures to support anthropogenic activities may create novel conditions that are exploited by invading species [21], [22] and our surveys of the vertical surfaces of pilings, pontoons and natural reefs provide evidence that non-indigenous species can dominate artificial structures in the marine environment. This agrees with previous research by Glasby et al. [29] and we identified non-indigenous invertebrates as the primary drivers of this pattern.

Urbanisation and the associated modification of habitats results in localized changes to abiotic and biotic conditions. For example, altered climates and habitats in cities relative to undeveloped areas can be exploited by exotic plants and animals [40]–[42]. In the marine environment, the construction of artificial structures also modifies local environmental conditions and we identified shading from these structures as an important factor in non-indigenous invertebrate recruitment. Shading in the marine environment alters light and sedimentation and these changes in environmental conditions likely play a major role in invertebrate recruitment since low levels of sedimentation reduces the chance that recruits will be smothered or that sediment will clog the filter feeding appendance of adult sessile inverts [43]. Similarly, low light levels under artificial structures can reduce algal cover and enhance invertebrate recruitment [32], [44]. Since the majority of non-indigenous species identified in this study were invertebrates, abiotic conditions associated with artificial structures (e.g. light and sedimentation) have the potential to create an ideal habitat for invading species. However, our results suggest that additional biotic and abiotic factors may also be important.

Previous experimental work has demonstrated that abiotic conditions can be the most important factors influencing the invasibility of a system [45]. Invasions in urbanized areas have been linked to the provision of habitat and refuges [42], [46], [47]. Non-indigenous invertebrate distributions on our experimental “reefs” suggest that orientation is one of the most important factors influencing the invasibility of shallow estuarine habitats. The orientation of experimental “reefs” explained most of the variation in overall community composition with non-indigenous invertebrates negatively affected by sedimentation on horizontal surfaces. Native invertebrates also appeared negatively affected by sedimentation. These effects are consistent with other studies which have found sedimentation to limit invertebrates relative to algae, regardless of whether they are native or non-indigenous [32], [33], [35], [36], [48], [49]. However, native invertebrates were significantly more successful than non-indigenous invertebrates on horizontal reefs and this could potentially be related to differential evolutionary tolerances. For example, non-indigenous invertebrates adapted to transport on ship hulls are not likely to be adapted to high sediment loads on horizontal surfaces, while native invertebrates may be better adapted to local environmental conditions [50]. We also found that native invertebrate recruitment was greatest on vertical shaded “reefs” and covers were negatively related to light availability suggesting that shading and orientation are important factors affecting their ability to colonise an established assemblage. Native algal covers were greatest on unshaded plates (with light levels similar to natural reefs). Since algae and invertebrates are in direct competition for space [51], it appears that changes in abiotic conditions on reefs have the potential to control the identity of the resident community, for example if a reef became shaded by an artificial structure.

Previous research has highlighted the abiotic and biotic characteristics that differ between artificial and natural habitats [27] and has provided evidence that these characteristics contribute to differences in their respective resident communities [52]. Comparisons between our survey and experimental work suggest that additional factors may contribute to the differential invasibility of artificial and natural habitats. We found substantially greater cover of non-indigenous invertebrates on pilings and pontoons (∼30–70%) compared to reefs and these covers could not be replicated with shading or orientation manipulations of experimental “reefs”. However, the covers of non-indigenous and native invertebrates found on natural reefs were replicated by the experimental “reefs” (around 5–12% covers). This suggests that reduced light and sedimentation under artificial structures are important, but not the only drivers of invasibility despite influencing invertebrate abundance [32], [33], [35]. Successful invasions have been linked to propagule pressure and artificial structures are often situated in close proximity to boat hulls which provide an ongoing supply of propagules that may be entrained within a marina [53]. This and the tendency for many sessile species to settle in proximity to conspecifics [54] may result in few invaders dispersing from artificial structures to reefs.

Terrestrial and marine urbanization results in the creation of islands of artificial structures and habitats that are surrounded by natural habitat [40]. This anthropogenic fragmentation of habitats may increase connectivity, and therefore aid invader spread [55]–[57]. These artificial islands are generally more heavily invaded than their natural surroundings [30], [40], and dispersal from artificial habitats is reliant on both abiotic and biotic conditions that contribute to the invasibility of natural habitats [4], [9], [58]. Marine artificial structures and reefs differ in their proximity to the benthos and are likely to experience different levels of scouring by sediment. Artificial structures may also be composed of different substrata to natural habitats. These factors can contribute to differing hard substrate assemblages [52], [59]–[62] and also affect the invasibility of habitats [28], [63], [64]. NIS exhibit some preference for shallow floating structures such as pontoons [28], [29], [65], potentially because they present a similar surface to a vessel hull with respect to movement and depth. Hull fouling has been recognized as a major source of invaders [66]–[69], and those arriving on vessels will likely have been selected for their preference to settle on shallow floating surfaces. However, this study was unable to detect conclusive differences between non-indigenous invertebrate diversity on pilings and pontoons. Differences in distribution between artificial and natural structures were often particular to one site or one sampling time. This was potentially due to differences in the existing assemblages at each location, the substrate composition between marinas [62], [64] as well as historical cleaning practices on pilings and pontoons which would have added to general variability.

Functional similarities between invading species and the native species in the recipient community may also influence invasibility. Examples from the literature suggest that where non-indigenous species share similar traits with the resident native species, they may be able to co-exist or competitively exclude their native counterpart [70], [71]. Sessile invertebrates (colonial and solitary) were an important functional group in our study and non-indigenous and native representatives were similarly distributed between artificial structures and reef walls. Manipulations of experimental “reefs” enhanced this observation with evidence that non-indigenous and native invertebrates exhibited similar responses to abiotic conditions (e.g. orientation). Algae were also a dominant group and in contrast to the invertebrates, native and non-indigenous representatives differed significantly in their ability to occupy space on artificial structures and natural reefs. Native algae were more successful in all habitats, while non-indigenous algae appeared to follow a “boom and bust” pattern of colonisation, differing between sites and sampling times. Our survey work found non-indigenous algae were present on apparently undisturbed horizontal reefs so they are able to invade established native assemblages. However, in experimental “reefs” deployments non-indigenous algae (*C. sinuosa* in our study system) recruited only to vertical surfaces that were bare, suggesting that they may be inhibited by the presence of a resident assemblage. While the non-indigenous algae in the current study do not appear to be particularly invasive, there are numerous examples of destructive macroalgal invasions in various parts of the world (reviewed by [72]) e.g. *Undaria pinnatifida* [73] and *Codium fragile* ssp. *fragile* (Suringar) Hariot [24] and where present on artificial structures they should be considered high-risk for spread due to functional similarities to rocky reef algal communities [73] [24].

Grassland studies suggest resource limitation (e.g. space and nutrients) must be overcome for successful invasion [71]. The release of resources such as nutrients enhances growth and therefore the potential for invaders to reach reproductive maturity faster [9]. While there is little evidence in rocky reef systems for nutrient limitation, estuaries that have artificially enhanced nutrient levels might be subject to increased invasion risk. The availability of primary space is more widely recognized as the most important limiting resource for marine sessile organisms [74], and space made available by disturbance to natural reef or provision of new surfaces on artificial structures has been linked to successful invasion by the algal species *Undaria pinnatifida* and *Codium fragile* ssp. *fragile* (Suringar) Hariot [23], [24], [73], [75].

Resource availability may also limit the establishment and spread of invasive species if the invading species are not able to out-compete resident native species [9]. Terrestrial studies have cited several examples where invasive species have been urban-adapted and are able to competitively exclude their native counterparts [76] or more competitive non-indigenous species [46], but our study suggests the opposite is occurring in the marine subtidal rocky reef environment. Community ecology from coastal rocky reefs predicts that native algae will dominate well-lit horizontal reefs while native sessile invertebrates are more abundant on shaded vertical reefs [35], [44]. However, we found that invertebrates (non-indigenous and native) occupied little space on horizontal or vertical reefs when an established assemblage was present, which could indicate competitive exclusion by the resident algal assemblage. Native algae in our study had similar covers on horizontal and vertical reefs and were similarly distributed on the experimental “reefs”. These results probably reflect the different conditions available to hard-substrate algae and invertebrates between open coastal reefs and the shallow estuarine reefs where our surveys and experiments were conducted. In shallow estuarine reef systems both the horizontal and vertical surfaces are well lit and close to the benthos creating optimal conditions for algal recruitment. Systems where the resident community is represented by a spatially dominant algal cover experience reduced invasibility as a result of biotic resistance [73]. The functional composition of algal assemblages has also been found to differentially limit colonization (encrusting algal species) and survivorship (canopy forming algal species) [4]. We found that the initial resident assemblage of our experimental ‘reefs’ (*Sargassum vestitum* canopy) played a role in reducing invisibility. However, the provision of experimental “reefs” that lacked a starting assemblage only slightly enhanced covers of non-indigenous invertebrates compared to what was observed on natural reefs in the initial survey. Moreover, non-indigenous invertebrate covers on bare experimental “reefs” were not much greater than on experimental “reefs” that had a resident assemblage. Together this suggests that biotic resistance in the form of competitive exclusion is not the primary factor controlling invasibility in our reef system.

Predation is often cited with competition as an important factor providing biotic resistance to invasion [77]. In the shallow rocky reef system, mobile micro-predators in the turfing algae and local fish species may provide biotic resistance against non-indigenous species colonisation [59], [78]. Experimental manipulations have demonstrated strong negative effects of native predators on invasion [7] and survey work suggests that a major difference between artificial structures and natural habitats are the availability and diversity of local predators [79]. Therefore differential predation could go some way to explaining why many invaders are absent from the reef but abundant on artificial structures only 10 s of metres away. Some native predator abundances are reduced in urban areas [80], and this may also contribute to the greater incidence of invasion in cities compared to surrounding rural areas through differential biotic resistance. However there is also evidence of increased non-native predators in urban areas that create a predation risk for native and non-native birds [81]. Although not investigated in the current study, future experiments comparing artificial structures and natural reefs would benefit from detailed consideration of the role predators play in biotic resistance.

## Supporting Information

Figure S1 **Diagram of the experimental “reefs” deployment frame.**
(DOCX)

Table S1 **List of species found during survey and their classification status as native (N), non-indigenous (NIS), cryptogenic or unidentified (C).** *D. listerianum* **has been classified as NIS or C by different authors and in this study was treated as NIS following the reasoning of Ruiz et al. (2000).**
(DOCX)

Table S2 **Mean ± S.E. percent covers of cryptogenic species from the survey of artificial and natural habitats and the experimental “reef” deployments.**.
(DOCX)
