# Supplementary material for: Comparing the Invasibility of Experimental “Reefs” with Field Observations of Natural Reefs and Artificial Structures
Source: PLoS One. 2012 May 30;7(5):e38124. doi: 10.1371/journal.pone.0038124 (PMC3364312; doi:10.1371/journal.pone.0038124)
Supplement: Figure S1 — Diagram of the experimental “reefs” deployment frame. (DOCX) [file pone.0038124.s001.docx]

Figure S1. Diagram of the experimental “reefs” deployment frame.
