# Supplementary material for: Comparing the Invasibility of Experimental “Reefs” with Field Observations of Natural Reefs and Artificial Structures
Source: PLoS One. 2012 May 30;7(5):e38124. doi: 10.1371/journal.pone.0038124 (PMC3364312; doi:10.1371/journal.pone.0038124)
Supplement: Table S1 — List of species found during survey and their classification status as native (N), non-indigenous (NIS), cryptogenic or unidentified (C). D. listerianum has been classified as NIS or C by different authors and in this study was treated as NIS following the reasoning of Ruiz et al. (2000). (DOCX) [file pone.0038124.s002.docx]

#### **Table S1.** List of species found during survey and their classification status as native (N), non-indigenous (NIS), cryptogenic or unidentified (C). *D. listerianum* has been classified as NIS or C by different authors and in this study was treated as NIS following the reasoning of Ruiz et al. (2000).

| **Group** | **Status** | **Native Distribution** | **Source** |
| --- | --- | --- | --- |
| **Algae** |  |  |  |
| *Colpomenia sinuosa* | NIS | Unknown | [[1](#_ENREF_1)] |
| *Dictyota dichotoma* | NIS | Unknown | [[1](#_ENREF_1)] |
| *Ecklonia radiata* | N |  |  |
| Encrusting algae (dominated by *Peyssonnelia* sp.) | N |  | [[2](#_ENREF_2)] |
| *Padina fraseri* | N |  |  |
| *Sargassum* vestitum | N |  | [[3](#_ENREF_3),[4](#_ENREF_4),[5](#_ENREF_5)] |
| Turfing algae (dominated by *Corallina officinalis* and to a lesser extent *Champia viridis* and *Laurencia* sp.) | N |  | [[2](#_ENREF_2)] |
| **Arthropoda** |  |  |  |
| *Amphibalanus variegatus* | N |  |  |
| *Balanus trigonus* | N |  |  |
| **Cnidaria** |  |  |  |
| *Aiptasia* sp. | C |  |  |
| Anemone sp.1 | C |  |  |
| Anemone sp.2 | C |  |  |
| *Culicia tenella* | N |  |  |
| Hydroid sp.1 | C |  |  |
| Hydroid sp.2 | C |  |  |
| *Telesteo multiflora* | N |  |  |
| **Echinodermata** |  |  |  |
| *Anthenea sidneyensis* | N |  |  |
| *Centrostephanus rogersii* | N |  |  |
| **Ectoprocta** |  |  |  |
| *Beania magellanica* | N |  |  |
| *Bowerbankia gracilis*. | NIS | W Atlantic | [[6](#_ENREF_6),[7](#_ENREF_7)] |
|  |  |  |  |
| Bryozoan sp.1 | C |  |  |
| Bryozoan sp.2 | C |  |  |
| *Bugula flabellata* | NIS | NE Atlantic | [[7](#_ENREF_7),[8](#_ENREF_8)] |
| *Bugula neritina* | NIS | NE Atlantic | [[6](#_ENREF_6),[7](#_ENREF_7),[8](#_ENREF_8)] |
| *Bugula stolonifera* | NIS | Southern Britain | [[7](#_ENREF_7),[9](#_ENREF_9)] |
| *Celleporaria nodulosa* | N |  |  |
| *Conopeum seurati* | NIS | Mediterranean and NE Atlantic | [[7](#_ENREF_7),[8](#_ENREF_8)] |
| *Cryptosula pallasiana* | NIS | N Atlantic | [[7](#_ENREF_7),[10](#_ENREF_10)] |
| *Crisia acropora* | N |  |  |
| *Fenestrulina mutabilis* | N |  |  |
| *Microporella umbracula*. | NIS | Mediterranean, W Atlantic and Indo-Pacific? | [[11](#_ENREF_11)] |
| *Schizoporella errata* | NIS | Mediterranean | [[7](#_ENREF_7),[8](#_ENREF_8)] |
| *Scruparia* sp. | C |  |  |
| *Tricellaria inopinata* | NIS | NE and NW Pacific | [[7](#_ENREF_7),[8](#_ENREF_8)] |
| *Watersipora subtorquata* | NIS | Unknown | [[6](#_ENREF_6)] |
| *Watersipora arcuata* | NIS | Indo-West Pacific | [[6](#_ENREF_6)] |
| **Mollusca** |  |  |  |
| *Anomia trigonopsis* | N |  |  |
| *Mytilus galloprovincialis planulatus* | N |  |  |
| *Saccostrea glomerata* | N |  |  |
| **Polychaeta** |  |  |  |
| *Galeolaria caespitosa* | N |  |  |
| *Hydroides elegans* | NIS | Unknown | [[8](#_ENREF_8)] |
| *Ficopomatos enigmatus* | NIS | Unknown | [[7](#_ENREF_7)] |
| *Salmacina australis* | N |  |  |
| *Pomatoceros taeniata* | N |  |  |
| Spirorbidae | C |  |  |
| *Chaetopterus* sp. | C |  |  |
| **Porifera** |  |  |  |
| Sponge sp.1 | C |  |  |
| Sponge sp.2 | C |  |  |
| Sponge sp.3 | C |  |  |
| Sponge sp.4 | C |  |  |
| Sponge sp.5 | C |  |  |
| Sponge sp.6 | C |  |  |
| **Tunicata** |  |  |  |
| Ascidiella aspersa | NIS | NW Atlantic, Mediterranean | [[12](#_ENREF_12)] |
| Botrylloides magnicoecum | N |  |  |
| Botrylloides leachi | NIS | NW Pacific | [[8](#_ENREF_8)] |
| Botryllus schlosseri | NIS | NW Atlantic, NE Pacific, Australia, New Zealand | [[8](#_ENREF_8)] |
| *Ciona intestinalis* | NIS | Atlantic | [[6](#_ENREF_6)] |
| *Didemnid* sp. | C |  |  |
| *Diplosoma listerianum* | NIS | NW Pacific | [[6](#_ENREF_6),[13](#_ENREF_13)] |
| *Herdmania momus* | N |  |  |
| *Perophora japonica* | NIS | Japan, Korea | [[14](#_ENREF_14),[15](#_ENREF_15)] |
| *Pyura stolonifera* | N |  |  |
| Solitary ascidian sp.1 | C |  |  |
| Solitary ascidian sp.2 | C |  |  |
| Solitary ascidian sp.3 | C |  |  |
| *Styela plicata* | NIS | East Asian Seas | [[7](#_ENREF_7)] |
|  |  |  |  |

References

1. Lewis J (1999) A review of the occurrence of exotic macroalgae in Southern Australia, with emphasis on Port Phillip Bay, Victoria. The Introduced Species of Port Phillip Bay, Victoria. Hobart: CSIRO Marine Research. 61-87 p.

2. Glasby TM, Connell SD, Holloway MG, Hewitt CL (2007) Nonindigenous biota on artificial structures: could habitat creation facilitate biological invasions? Marine Biology 151: 887-895.

3. Agardh JG (1889) Species Sargassorum Australiae descriptae et dispositae Kongl. Svenska Vetenskapsakademiens Handlingar 23: 1-133.

4. Millar AJK, Kraft GT (1994) Catalogue of marine brown algae (Phaeophyta) of New South Wales, including Lord Howe Island, South-western Pacific. Australian Systematic Botany 7: 1-46.

5. Guiry MD, Guiry GM (2012) AlgaeBase. World-wide electronic publication, National University of Ireland, Galway.

6. Ruiz GM, Fofonoff PW, Carlton JT, Wonham MJ, Hines AH (2000) Invasion of coastal marine communities in North America: apparent patterns, processes and biases. Annual Review of Ecology and Systematics 31: 481-531.

7. Keough MJ, Ross DJ (1999) Introduced fouling species in Port Phillip Bay. In: Hewitt C, Campbell M, Thresher R, Martin R, editors. The Introduced Species of Port Phillip Bay, Victoria Centre for Research on Introduced Marine Pests Technical Report No 20. Hobart: CSIRO Marine Research. pp. 193-229.

8. Hewitt C, Campbell M, Thresher R, Martin R, Boyd S, et al. (2004) Introduced and cryptogenic species in Port Phillip Bay, Victoria, Australia. Marine Biology 144: 183-202.

9. USGS (2008) United States Geological Survey: *Bugula stolonifera*. Date of access: 30-Jun-2009.

10. NIMPIS (2002) *Cryptosula pallasiana* species summary. . In: Hewitt CL, Martin RB, Sliwa C, McEnnulty FR, Murphy NE et al., editors. National Introduced Marine Pest Information System Web publication <[http://crimpmarinecsiroau/nimpis>](http://crimpmarinecsiroau/nimpis%3e). Date of access: 11-Nov-2006.

11. Audouin JV (1826) Prodrome d'une histoire naturelle, chimique, pharmaceutique, et medi-cale des cantharides. Paris.

12. NIMPIS (2012) *Ascidiella aspersa* species summary. . In: Hewitt CL, Martin RB, Sliwa C, McEnnulty FR, Murphy NE et al., editors. National Introduced Marine Pest Information System Web publication <[http://wwwmarinepestsgovau/nimpis>](http://wwwmarinepestsgovau/nimpis%3e). Date of access: 21-Mar-2012.

13. Lambert CC, Lambert G (1998) Non-indigenous ascidians in southern California harbors and marinas. Marine Biology 130: 675-688.

14. Lambert G (2005) First North American record of the ascidian *Perophora japonica*. Journal of the Marine Biological Association of the United Kingdom 85: 1011-1012.

15. Nishikawa T, Bishop JDD, Sommerfeldt AD (2000) Occurrence of the alien ascidian *Perophora japonica* at Plymouth. Journal of the Marine Biological Association of the United Kingdom 80: 955-956.
