# Supplementary material for: Comparing the Invasibility of Experimental “Reefs” with Field Observations of Natural Reefs and Artificial Structures
Source: PLoS One. 2012 May 30;7(5):e38124. doi: 10.1371/journal.pone.0038124 (PMC3364312; doi:10.1371/journal.pone.0038124)
Supplement: Table S2 — Mean ± S.E. percent covers of cryptogenic species from the survey of artificial and natural habitats and the experimental “reef” deployments.. (DOCX) [file pone.0038124.s003.docx]

Table S2. Mean ± S.E. percent covers of cryptogenic species from the survey of artificial and natural habitats and the experimental “reef” deployments.

| **Cryptogenic species** | **Survey** | **Experimental "reef"** |
| --- | --- | --- |
| *Aiptasia* sp. |  | 1.42±0.17 |
| Anemone sp.1 | 0.02±0.02 |  |
| Anemone sp.2 |  | 0.12±0.04 |
| Hydroid sp.1 |  | 0.05±0.02 |
| Hydroid sp.2 | 0.48±0.11 |  |
| *Scruparia* sp. |  | 1.05±0.19 |
| Bryozoan sp.1 |  | 0.02±0.01 |
| Bryozoan sp.2 |  | 0.01±0.01 |
| Spirorbidae | 0.05±0.03 | 3.2±0.46 |
| Sponge sp. 1 | 0.12±0.06 | 0.62±0.14 |
| Sponge sp.2 | 2.65±0.33 |  |
| Sponge sp. 3 | 0.19±0.05 |  |
| Sponge sp. 4 | 0.86±0.27 |  |
| Sponge sp. 5 | 0.25±0.09 |  |
| Sponge sp. 6 | 0.1±0.07 |  |
| Didemnid sp. | 0.01±0.01 |  |
| Solitary ascidian sp.1 |  | 1.58±0.29 |
| Solitary ascidian sp.2 |  | 0.07±0.07 |
| *Chaetopterus* sp. |  | 2.11±0.38 |
|  |  |  |
